# Clinical T Cell Receptor Repertoire Deep Sequencing and Analysis: An Application to Monitor Immune Reconstitution Following Cord Blood Transplantation

**DOI:** 10.3389/fimmu.2018.02547

**Published:** 2018-11-05

**Authors:** Athina Soragia Gkazi, Ben K Margetts, Teresa Attenborough, Lana Mhaldien, Joseph F. Standing, Theres Oakes, James M. Heather, John Booth, Marlene Pasquet, Robert Chiesa, Paul Veys, Nigel Klein, Benny Chain, Robin Callard, Stuart P. Adams

**Affiliations:** ^1^Infection, Immunity and Inflammation Section, Great Ormond Street Institute of Child Health, University College London, London, United Kingdom; ^2^Digital Research Environment, Great Ormond Street Hospital for Children NHS Foundation Trust, London, United Kingdom; ^3^Centre for Computation, Mathematics, and Physics in the Life Sciences and Experimental Biology (CoMPLEX), University College London, London, United Kingdom; ^4^SIHMDS-Haematology, Great Ormond Street Hospital for Children NHS Foundation Trust, London, United Kingdom; ^5^Pharmacy Department, Great Ormond Street Hospital for Children NHS Foundation Trust, London, United Kingdom; ^6^Division of Infection and Immunity, University College London, London, United Kingdom; ^7^Le Centre Hospitalier Universitaire de Toulouse, Toulouse, France; ^8^Department of Blood and Marrow Transplantation, Great Ormond Street Hospital for Children NHS Foundation Trust, London, United Kingdom; ^9^Infectious Diseases Department, Great Ormond Street Hospital for Children NHS Foundation Trust, London, United Kingdom

**Keywords:** T cell, haematopoietic stem cell transplant, next generation sequencing, T cell receptor, CDR3, clonotypes, immune reconstitution

## Abstract

Spectratyping assays are well recognized as the clinical gold standard for assessing the T cell receptor (TCR) repertoire in haematopoietic stem cell transplant (HSCT) recipients. These assays use length distributions of the hyper variable complementarity-determining region 3 (CDR3) to characterize a patient's T cell immune reconstitution post-transplant. However, whilst useful, TCR spectratyping is notably limited by its resolution, with the technique unable to provide data on the individual clonotypes present in a sample. High-resolution clonotype data are necessary to provide quantitative clinical TCR assessments and to better understand clonotype dynamics during clinically relevant events such as viral infections or GvHD. In this study we developed and applied a CDR3 Next Generation Sequencing (NGS) methodology to assess the TCR repertoire in cord blood transplant (CBT) recipients. Using this, we obtained comprehensive TCR data from 16 CBT patients and 5 control cord samples at Great Ormond Street Hospital (GOSH). These were analyzed to provide a quantitative measurement of the TCR repertoire and its constituents in patients post-CBT. We were able to both recreate and quantify inferences typically drawn from spectratyping data. Additionally, we demonstrate that an NGS approach to TCR assessment can provide novel insights into the recovery of the immune system in these patients. We show that NGS can be used to accurately quantify TCR repertoire diversity and to provide valuable inference on clonotypes detected in a sample. We serially assessed the progress of T cell immune reconstitution demonstrating that there is dramatic variation in TCR diversity immediately following transplantation and that the dynamics of T cell immune reconstitution is perturbed by the presence of GvHD. These findings provide a proof of concept for the adoption of NGS TCR sequencing in clinical practice.

## Introduction

Haematopoietic stem cells transplantation (HSCT) utilizes three different stem cell sources; Bone Marrow (BMT), Peripheral Blood (PBSCT) or Umbilical Cord (CBT). The stem cell choice usually depends on availability, underlying disease, and clinical status (1). Initial HSCTs were carried out using bone marrow as a stem cell source, but in 1989, Broxmeyer and colleagues demonstrated that cord blood has similar attributes to bone marrow and contains significant numbers of progenitor cells, and suggested cord blood as a possible alternative source to bone marrow in transplantation (2). The first successful CBT was performed by Dr. Eliane Gluckman of Hospital St. Louis, Paris, France in a 5-years-old boy suffering from Fanconi's Anemia (3). Since then CBT has been widely used as a treatment for many diseases, with results from the Cord Blood Transplantation Study (COBLT) demonstrating the high uptake of CBT in the US (4). Similarly, since the incorporation of CBT into standard practice in the UK was recommended (5, 6), the number of CBTs has increased (7).

CBT has been used with considerable success for the treatment of malignant diseases (8), primary immunodeficiencies (9), and metabolic disorders (10). Importantly, it has also been shown to result in less graft *vs*. host disease (GvHD) compared with adult BMT donors (allowing for the degree of HLA mismatch; (11)). Since the speed at which the immune system recovers after HSCT has a direct bearing on outcome (12) and given that CBT (with *in vivo* T cell depletion) has been shown to result in impaired T cell immune reconstitution (13), we recently demonstrated that the omission of serotherapy can lead to a rapid thymic-independent T cell expansion following CBT (14). These rapidly expanding naïve T lymphocytes, particularly CD4+ T cells, have generated considerable interest as it has been shown that they can differentiate into viral-specific T cells within 2 months and are able to clear viral infections (14). Rapid recovery of the CD4+ T cells is associated with less transplant related mortality (15). In addition, it has been demonstrated *in vitro* that CBT-derived T cells are able to mediate a more potent anti-leukaemic effect than adult T cells (16), which is also apparent *in vivo* in acute myeloid leukemia patients undergoing transplantation in the presence of minimal residual disease (17, 18). Since immune reconstitution following HSCT is so important for both a successful short-term and long-term outcome, with regards to GvL, GvHD, and response to viral infection, comprehensive methodologies are needed to better assess this process.

Measuring immune reconstitution following CBT using immunophenotyping with appropriate markers and using molecular quantification of T cell receptor excision circles (TRECs) has proved to be useful in assessing thymic-dependent and independent T cell recovery following CBT (14). Additionally, the assessment of the T cell receptor (TCR) repertoire has been carried out using T cell spectratyping (14). With the advent of Next Generation Sequencing (NGS) it is now possible to analyse the TCR repertoire in much greater depth to identify individual TCR clones and sequences (19, 20). We questioned whether this emerging NGS technology could be established to more efficiently provide TCR repertoire data from cord blood units used for CBT. We also aimed to develop methods for measuring TCR repertoire diversity and to identify relationships between these measurements and clinical outcomes following CBT; specifically antigen specificity, GvHD, and cell dosage.

## Methods

### Samples

This study involved the use of excess diagnostic blood taken from patients at Great Ormond Street Hospital and was anonymised prior to use. The study was approved by the National Research Ethics Service, NRES Committee London—Bloomsbury (05/Q0508/61) for “Cellular immune reconstitution following haematopoietic stem cell transplantation.” Thirty-nine samples were analyzed from 5 control cord samples (not used for transplantation in this study) and 16 Great Ormond Street Hospital (GOSH) patients. Additionally, due to the difficulty in obtaining age-matched pediatric controls, 58 adult control PBMC samples were analyzed for later comparison with control cord data. The median age at transplant was 2 years and 1 month and the patients ranged from 0.4 to 7.7 years old. Underlying conditions included a number of different primary immunodeficiencies (SCID, Wiscott Aldrich Syndrome, and MHC Class II deficiency), hematological malignancies (ALL, AML, and JMML) and one metabolic disorder (Hunter Syndrome). All patients were treated with allo-CBT and sampling was performed at multiple time points following transplantation (Table 1). Peripheral blood mononuclear cells were isolated from 10 ml of healthy adult volunteer blood. Samples were handled in accordance with the immune reconstitution study ethics attained at GOSH.

**Table 1:** Patient characteristics for all transplanted patients in the study.

**Patient**	**Disease**	**Age group at Tx (years)**	**Sampling Time Points (Months)**	**GvHD Grade**	**Conditioning**	**Mortality**
A	HM	1-2	2, 3, 6, 12	III	Myeloablative^1^	Deceased
B	PID	1-2	1, 2	I	Reduced Intensity	Alive
C	HM	2-5	0.5	III	Myeloablative^4^	Deceased
D	PID	0-1	1	I	Reduced Intensity	Alive
E	HM	0-1	1	II	Myeloablative^3^	Alive
F	PID	1-2	3, 19	I	Reduced Intensity	Alive
G	HM	1-2	1	II	Myeloablative^1^	Alive
H	PID	5-10	2, 6, 12	II	Reduced Intensity	Alive
I	HM	1-2	2, 4, 6, 12	IV	Myeloablative^1^	Alive
J	HM	5-10	1	IV	Myeloablative^3^	Deceased
K	HM	2-5	1, 3	0	Myeloablative^1^	Alive
L	PID	1-2	1, 2, 3, 12, 22	II	Reduced Intensity	Alive
M	PID	0-1	1, 2, 6, 22	II	Reduced Intensity	Alive
N	Metabolic	2-5	1, 3	0	Myeloablative^2^	Alive
O	HM	2-5	2, 12	II	Myeloablative^1^	Alive
P	PID	2-5	1, 2, 17, 30	II	Reduced Intensity	Alive

*“GvHD grade” refers to the maximum GvHD grade the patient exhibited. Patient ages at transplant were grouped into 0-1, 1-2, 2-5 or 5-10 years old. Sampling time points refers to the month post-CBT that the sample was taken on. Myeloablative conditioning comprised ofbusulfan/cyclophosphamide/melphalan^1^, busulfan/fludarabine^2^, treosulfan/fludarabine/thiotepa^3^ or busulfan/cyclophosphamide^4^. Reduced intensity conditioning consisted of treosulfan/fludarabine*.

### Immunophenotyping, spectratyping and TRECs

Routine immunophenotyping was carried out to measure T cell numbers and naïve/memory T cells as previously described (21) T cell receptor spectratyping (22) was carried out using the same starting RNA sample as for the NGS experiments. Quantification of TRECs was carried out on DNA extracted from the same starting sample as used for the NGS experiments and using previous published methods (21).

### Sample preparation

Peripheral Blood Mononuclear Cells (PBMCs) were isolated from the blood samples and RNA was extracted using the Qiagen RNA blood Mini kit (Qiagen, 52304). RNA was subsequently treated with RQ1 DNase (Promega) following manufacturer's instructions to remove any residual genomic DNA.

### Library preparation

Library preparation was based on a protocol for quantitative TCR sequencing. Full details of the method have been recently published (23).

### Library sequencing

Up to 12 final amplicon products were pooled together and loaded, at 12pM concentration, on an Illumina MiSeq, using a version 2 chemistry 2x250PE kit.

### Data analysis

The raw FastQ files are deposited at the Short Read Archive (https://www.ncbi.nlm.nih.gov/sra) under accession number SRP136075. Raw numbers of reads prior to subsampling are provided in Supplementary Table 1. The FASTQ files produced on the MiSeq were demultiplexed and UMI-corrected using an analysis pipeline that incorporates Decombinator (19, 23–25). All software is freely available at https://github.com/innate2adaptive/Decombinator.

Typically, when analyzing TCR data, the total number of TCRs in a sample are rarefied to either the minimum number of TCRs from all samples (or lower), or an arbitrarily selected percentage of the maximum read depth (usually 10%) of the sample containing the largest number of TCRs. Motivated by concerns over sample representation following rarefication, we developed an algorithm to compute the minimum representative subsampling depth for each sample (Supplementary Figure 1). To begin, rarefication curves were drawn for each sample between 100 and 1% of their total number of Decombinator-identified reads (DCRs), computing the Gini coefficient and Shannon entropy, normalized to their returned values at 100% read depth, at each 1% interval. The total population of DCRs in a sample was randomly subsampled 30 times for each iteration, and the mean and standard error calculated. The algorithm then computes the gradient of the mean normalized Gini coefficient decline at each subsampling depth, recording each event where the gradient >1.1, representing greater than linear depreciation over time, but not low enough to include minor variation. As the normalized Gini coefficient is bounded in x and y between 0 and 1, where subsampling will eventually tend toward a single sequence, giving an even population, and a returned Gini coefficient of 0, Gini coefficient was an ideal statistic to quantify the effect of rarefication on. The percentages in x where the gradient exceeded 1.1 were concatenated into arrays of continuous integers, with an order statistic assigned to each array, the lowest of which was selected. This assumes that the lowest continuous sequence of gradients above 1.1 would represent the point at which the distance between low frequency clonotypes and the top clonotypes reduce, confounding the quantifiable diversity in the sample. The largest subsampling value from this list + 1 was selected (i.e., a 20% subsample value would become 21%) and is marked as the minimum subsampling depth for that sample, the point just prior to where the gradient of mean normalized Gini coefficient decline exponentially increases. The maximum subsampling depth of a sample is inherently defined as equal to 100% of the DCRs identified in a sample. The algorithm then optimizes the greatest number of samples that can be included between their total number of DCRs, and their minimum subsampling depth, from which, for this study, a subsampling depth of 10,232 DCRs was determined.

Among the analyses applied to the data were two different diversity metrics, Gini coefficient and Shannon entropy. Gini coefficient is a measure of inequality, originally developed as an economics tool for measuring wealth distribution. It is classically defined as twice the area between the 45° -line and the Lorenz curve (a curve visualizing cumulative abundance against the cumulative population) for the data. The scale ranges between 0 and 1, where 0 = completely equal (x clones, all with identical frequencies) and 1 = completely unequal (i.e., tending toward sample oligoclonality). It can be used to gauge the inequality of the frequency distribution between different clonotypes in each sample, and give a summary of the clonotype abundance distribution.

Shannon entropy, a separate measure, quantifies diversity through the incorporation of both evenness and richness, with higher values representing more even populations of TCR rich samples. Shannon entropy, *H*(*X*), where *X* is the CDR3 data from a single sample, is defined as

(1) $$\begin{array}{r} H\left(X\right)=\;-\sum_{i=1}^{n}p\left(x_{i}\right)log_{b}^{*}\;p\left(x_{i}\right) \end{array}$$

where *p*(*x*_*i*_) is the proportional abundance of clonotype *i* and *b* is the logarithm base of 2.

For analyses, the *ineq* package (version 0.2.13) was used in the R environment (version 3.3.2) for computing the Gini coefficient along with the *vegan* package (version 2.4.2) for computing Shannon entropy. The *ggplot2* package (version 2.2.1) was used for all data visualizations. The subsampling algorithm and all manual data analyses were executed using custom scripts written in R. The Decombinator package v3.1 was executed using Python (version 2.7.3). The beta chain for each sample was used unless otherwise specified.

## Results

### Abnormal diversity tends toward normality following CBT

**Figure 1:**
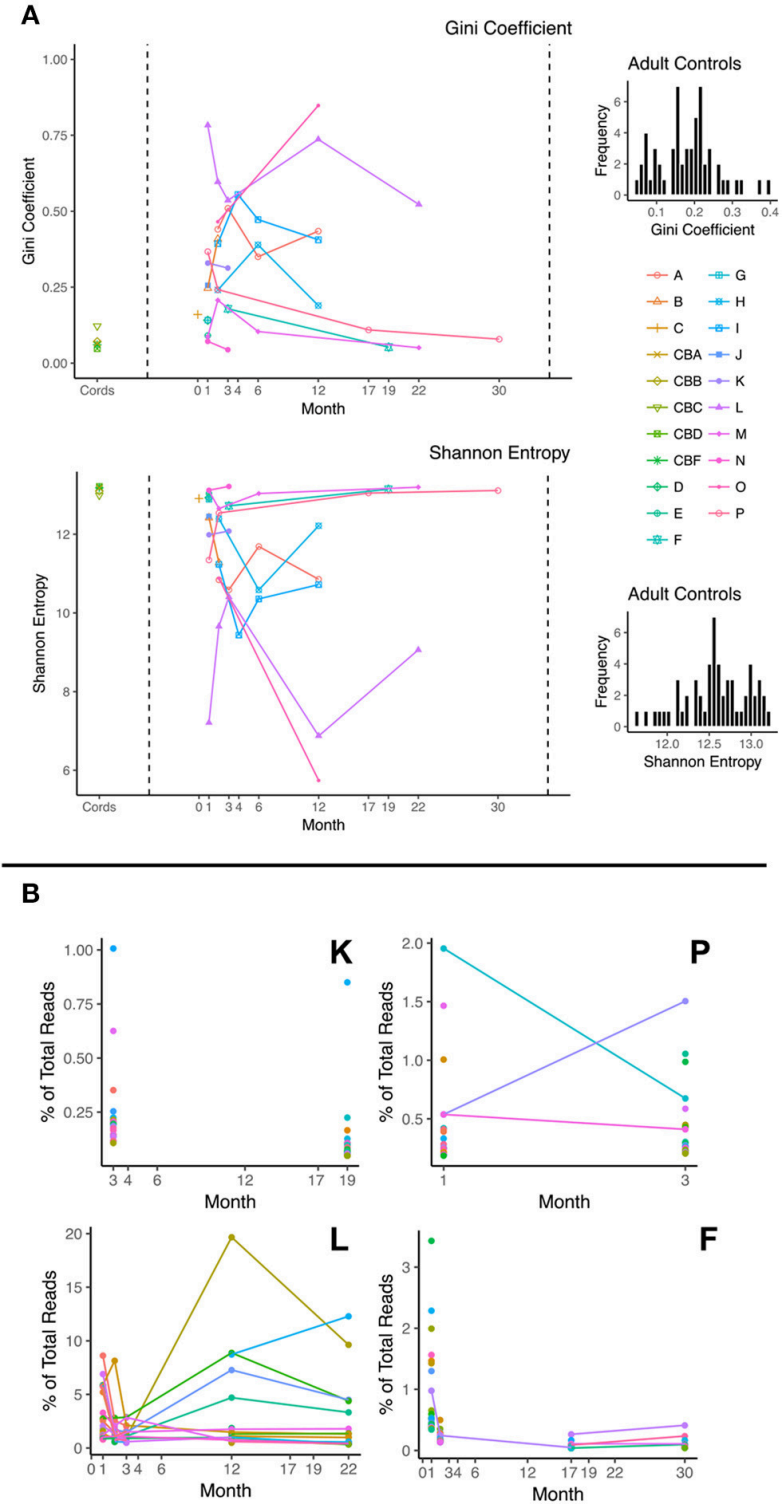
**(A)** Plots of Gini Coefficient and Shannon Entropy against month post-transplant; Diversity scores for control cord samples are shown on the left hand side and patient samples on the right. Each line represents a different patient. For comparison, adult control PBMC diversity histograms are presented next to these plots, demonstrating a similar diversity distribution to the control cord samples. **(B)** Frequency of most abundant 20 clonotypes as a function of time shown for 4 patients (K, P, L, and F). Each color represents a different clonotype. Lines between points represent a persistent clonotype detected at multiple time points.

We investigated TCR clonal distribution profiles using both the Gini coefficient (a measure of inequality) and the Shannon diversity index for all patients during immune reconstitution. The results for beta chain are displayed below (Figure 1A). We are able to demonstrate that the cord blood controls have a Gini coefficient < 0.2 and a Shannon diversity index of >12. By contrast many of the post-CBT patient samples exhibit higher Gini coefficients or lower Shannon diversity index values indicating an abnormal TCR repertoire. Diversity profiles are complex and exhibit multifactorial dependence on a large number of covariates following transplantation. The TCR repertoire becomes significantly more uneven (as demonstrated by increased Gini, and decreased Shannon) post-transplant, compatible with significant clonal expansion and repertoire dysregulation. There is a trend toward a subsequent fall in Gini index beyond 6 months post-transplant. However, the most striking feature of the data is the variance in both indices, both between and within patients, presumably reflecting major dynamic changes in repertoire composition post-transplant. As can be seen from Figure 1A, the majority of profiles exhibit trends toward normality over time, as defined by the diversity shown in the control cord population. Adult control PBMC data demonstrated a similar distribution of diversity values to control cords. This made cord data an excellent stand-in for data from age-matched control PBMCs. Patient O was the only patient to require a second graft (due to disease relapse and dropping chimerism) 6 months after the initial CBT. As can be seen, the trajectory of their profile appears to be abnormal in comparison with the other patient's profiles. Figure 1B also displays the most abundant 20 clonotypes as a percentage of total DCRs at different timepoints for a selection of patients, demonstrating the dynamic nature of the repertoire's clonal composition during a patient's recovery.

### Variance in TCR diversity is inherently linked with thymic output

**Figure 2:**
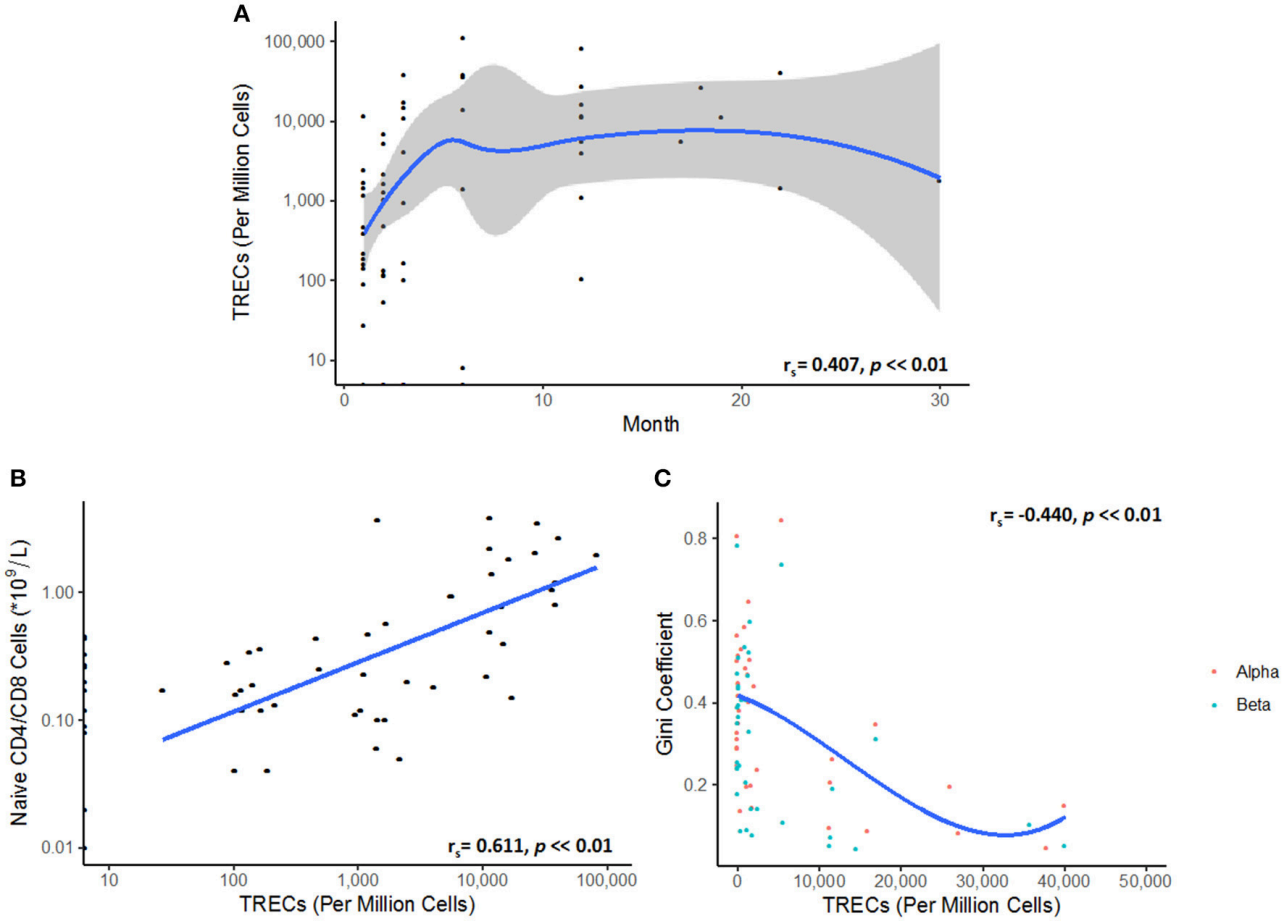
**(A)** TREC numbers rise and then plateau following transplantation. A Spearman's rank correlation coefficient test showed a partially monotonic relationship between TREC numbers and time after transplant (r_s_ = 0.407, *p* < < *0.01*). The data was fitted by LOESS regression (blue line). Confidence intervals are represented by the gray shaded area. Each point represents a single TREC count. **(B)** Naive T cell numbers correlate to TREC levels (r_s_ = 0.611, *p* < < *0.01*). **(C)** Repertoire diversity, as represented by the Gini coefficient, reflects the replenishment of the peripheral naïve repertoire via thymic export. A cubic spline model was fitted to the data, demonstrating the relationship between TRECs and TCR diversity (r_s_ = −0.440, *p* < < *0.01*).

The levels of thymic output, as measured by TRECs, are low in the initial stages of reconstitution, and then follow a non-linear logistic growth dynamic which plateaus between 4 and 6 months, reflecting a limit on thymic export, and the generation of new naïve TCRs (Figure 2A). As expected, the number of naïve T cells in blood is correlated to the number of TRECs (Figure 2B). Interestingly, the Gini index is inversely correlated to the number of TRECs, presumably reflecting the increase in repertoire diversity as more naïve cells enter the circulation (Figure 2C).

### GvHD and cell counts are correlated with TCR diversity, but not cell dose

**Figure 3:**
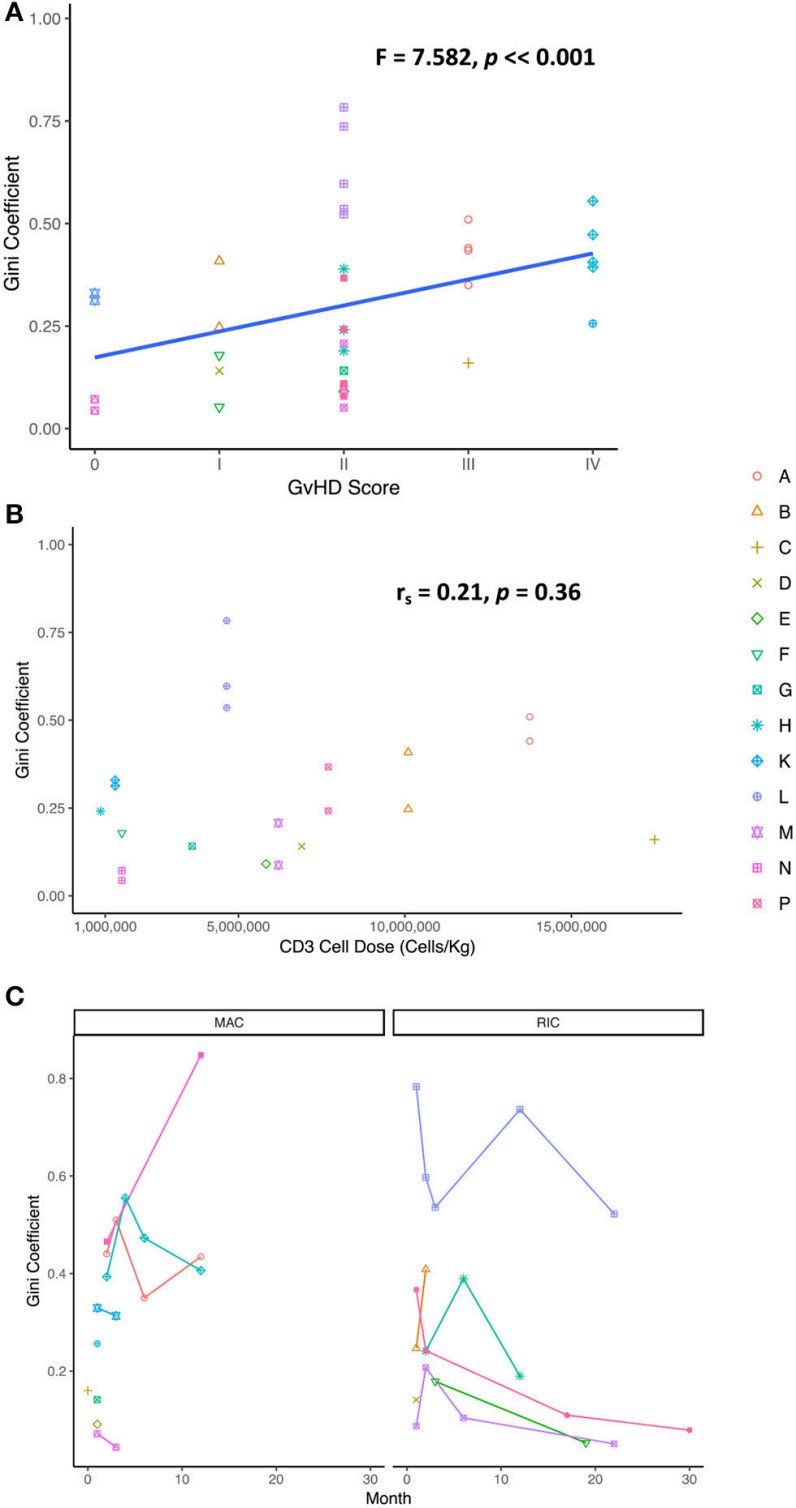
**(A)** GvHD score extracted from clinical notes found to correlate with TCR diversity (Gini coefficient) demonstrating a possible link between degree of sample clonality and severity of GvHD [one way ANOVA (*F* = 7.582, *p* = *0.00746*)]. **(B)** The Gini coefficient as a function CD3 cell dose in the cord blood unit [Spearman's rank correlation coefficient (r_s_ = 0.2086, *p* = *0.364*)]. **(C)** No correlation was observed between the Gini coefficient and the conditioning regimen following transplantation [Wilcoxon rank sum test (W = 221, *p* = *0.3791*)]. MAC, myeloablative conditioning; RIC, reduced intensity conditioning.

To understand the relationship between TCR repertoire reconstitution and the clinical parameters of reconstitution, we examined the Gini index as a function of level of graft mismatch, the conditioning regimen, the cell dosage per kg, severity of graft vs. host disease (GvHD), the presence of post-transplant infections, cell counts during reconstitution, and duration of any post-transplant immunosuppression. Interestingly, we found that diversity was inversely related to GvHD scores, suggesting that GvHD may be driving clonal expansions and hence reducing clonal diversity (Figure 3A). No significant correlation could be observed with any other clinical parameter. Specifically, we were unable to see any influence of the CD3+ cell dose given in the cord blood unit nor the conditioning regimen on diversity scores (Figures 3B,C).

### Verification of the antigen specificity

**Figure 4:**
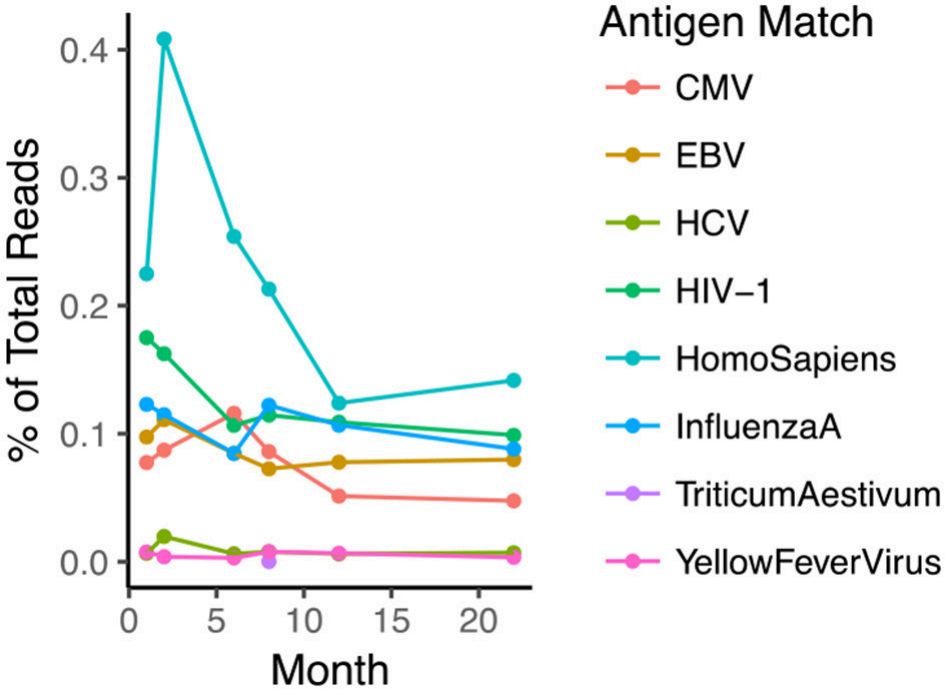
TCR sequences with previously described antigen-specific annotation are observed in the repertoire of the reconstituting patients. The plot shows a representative example of several annotated sequences in one patient in relation to time post-transplant (eight target species from which antigen peptide is derived is shown in Legend). Note that the patient was confirmed to be HIV-1 negative.

VDJdb (26) is an open-access TCR database that contains CDR3s annotated for antigen specificity. We investigated whether antigen specificity could be defined for the unique clonotypes in each sample, using VDJdb. We found several examples of TCR sequences from the database in the repertoires of the patients in this study (Figure 4). These shared sequences are likely to represent “public” TCRs which may have higher probabilities of being generated owing to the non-uniform nature of the recombination process (27). Unexpectedly, clonally expanded HIV-1 “specific” sequences were found in each reconstituted repertoire (ranging between 0.04–0.45% across all 16 CBT patients), despite all patients being confirmed to be HIV negative prior to transplantation. These observations suggest that the antigen specific annotations of these sequences should be treated with caution, as different pairing between alpha and beta chains, and cross-reactivity may both contribute to the antigen specificity of individual cells.

### Recovering and quantifying spectratyping classifications using TCR sequencing

**Figure 5:**
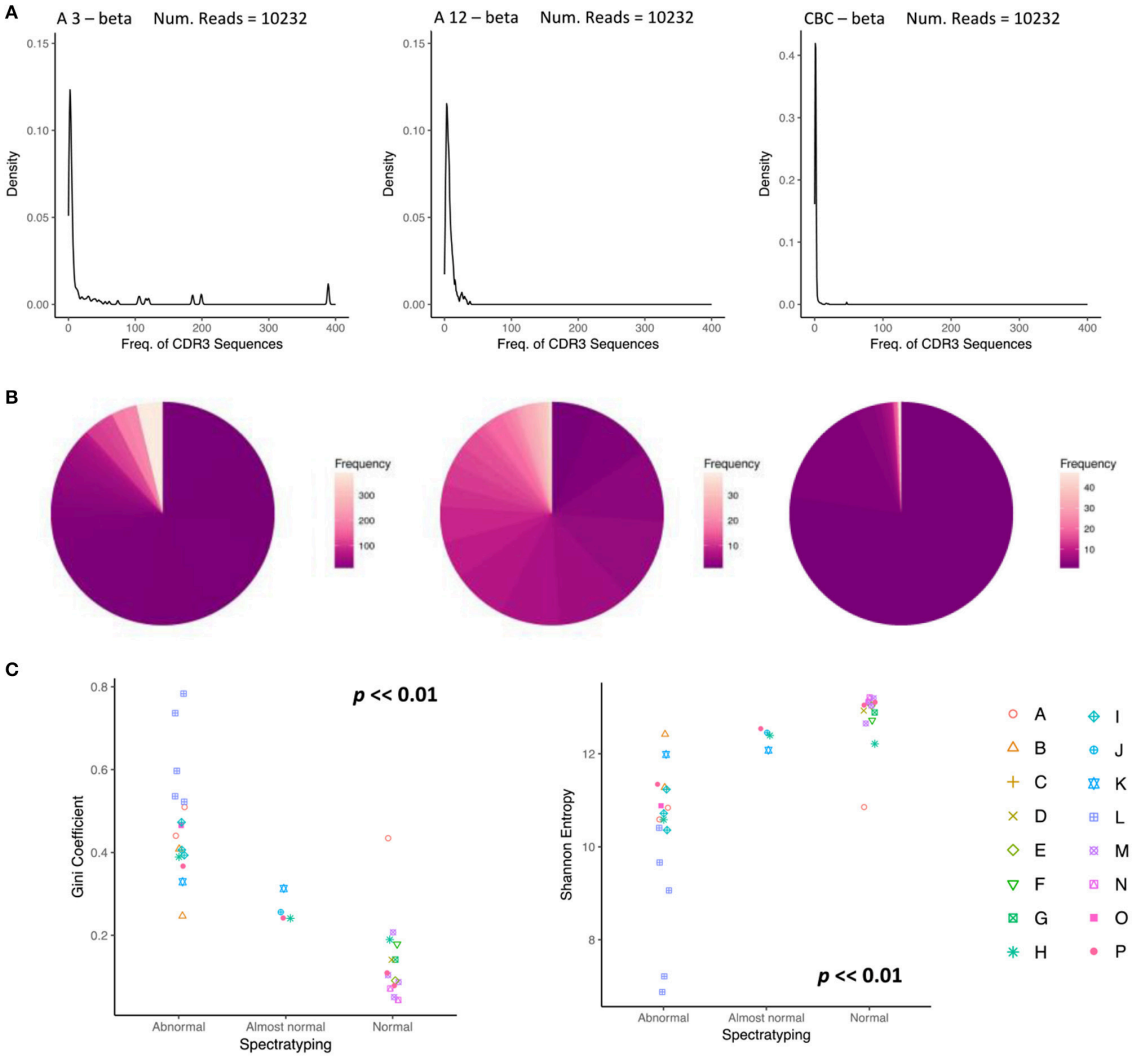
Changes in the abundance profile of CDR3 sequences reflect normalization of the repertoire. **(A)** A density plot showing the distribution of abundances for the beta chain of CBT patient A, (left), 3 months after transplant, classified as abnormal by spectratyping, (middle) 12 months after transplant, classified as normal by spectratyping, (right) and a control cord. At month 3, CDR3 frequency density profile is highly abnormal with an overrepresentation of clonal expansions. By month 12, the repertoire becomes much more even reflecting a shift back towards normality. **(B)** A pie chart reflecting the same clonotype expression profile as **(A)**, where color represents clonotype frequency. **(C)** TCR repertoire diversity reproduces the repertoire classification determined by spectratyping. Gini coefficient (left) and Shannon entropy (right) for each sample is plotted in relation to spectratyping classification.

The status of the reconstitution of the T cell compartment is currently investigated, inter alia, by classifying the “normality” of the repertoire using spectratyping. We investigated whether similar classification could be carried out on the basis of the TCR repertoire as assessed by NGS. Spectratypes are usually assessed utilizing both the median number of peaks per TCRVβ family and the number of TCRVβ families with a distribution that take an approximate Gaussian form. On the basis of these parameters, repertoires are assigned to three groups, “Abnormal,” “Almost normal” and “Normal.” We compared the abundance profile of two repertoires, classified by spectratyping as “Abnormal” (taken from a patient three months post-transplant) and “Normal” (taken from the same patient after 12 months) with a control cord sample (CBC) (Figures 5A,B). The three repertoires can readily be differentiated from each other. The 3 months “Abnormal” profile contains numerous high abundance clonal expansions dominating a portion of the TCR repertoire. The profile at 12 months is more evenly distributed, lacks drastically overrepresented clonotypes, and the overwhelming majority of sequences are observed < 10 times. Similarly, the control cord sample, CBC, expresses a comparable repertoire distribution to the spectratype-defined “Normal” sample. We next plotted diversity metrics obtained from all our samples, as a function of clinical spectratyping score (Figure 5C). The Gini and Shannon index were both significantly associated with spectratyping score (Kruskal-Wallis test, *p* < < *0.01*), suggesting that the diversity of the repertoire could contribute to immunophenotyping in assessing T cell reconstitution following transplantation in children (Figure 5C).

**Figure 6:**
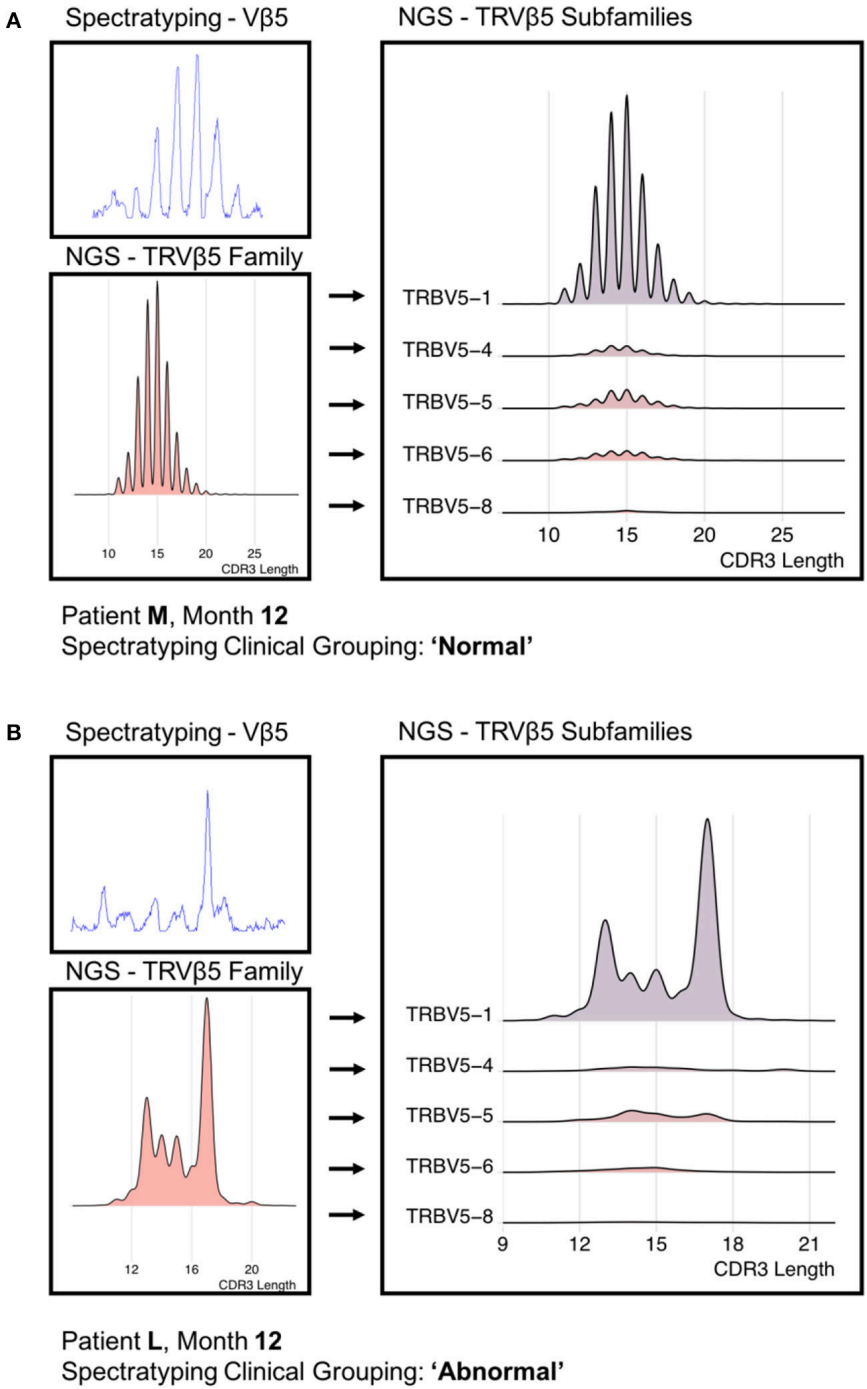
A “Normal” **(A)** PCR-derived spectratype from patient M and a “Abnormal” **(B)** PCR-derived spectratype from patient L (both 12 months post-transplant) compared to reconstructed spectratypes from time-matched NGS data. Due to the resolution offered by the NGS data, we were able to separate the NGS-derived spectratype into its constituent subfamilies.

Finally, we reconstructed “virtual” spectratype profiles by plotting the CDR3 length profile taken from the sequence data, and compared this to spectratypes from the same profile. A representative example of a “normal” and “abnormal” spectratype sample are shown in Figure 6. The virtual profiles were strikingly similar to those obtained by the classical protocol, and clearly demonstrated the differences between normal and abnormal repertories. However, the virtual spectratypes showed greater resolution, and additionally allowed analysis via V region subtype, which was not possible using the standard method. Our NGS implementation emphasizes the dominance of specific subfamilies in defining the distribution seen in a traditional spectratype.

## Discussion

We have developed a novel tool using NGS with an analytical pipeline and diversity scoring metrics to accurately quantify TCR diversity. We applied this to a cohort of patients undergoing CBT and have presented several findings that could not be seen using conventional TCR spectratyping techniques. Spectratyping, the widely-adopted clinical gold standard for T cell repertoire immunophenotyping, has been used since 1994 (28) with great success in monitoring the immune response, especially in patients following transplantation. Despite the technique's popularity, there are clear limitations in the data it produces, which stem from the era in-which it was developed. Spectratyping data are low-resolution depictions of the TCR repertoire, condensing complex immunological information into singular points describing CDR3 length, frequency, and associated gene. This inherently restricts the potential of the biological information available for extraction with modern methodologies. One pivotal issue is the lack of ability to deconvolve the TCR V gene family distributions into their clonotype constituents, which may or may not be made up of multiple antigen-distinct clonotypes. An emerging methodology that addresses these limitations is TCR repertoire sequencing, such as the protocol utilized in this study (23). Other protocols have been recently developed to quantitatively analyse the T cell repertoire in patients post-HSCT, but this has so far been limited to either adult recipients (29, 30), or restricted to beta chain analysis only (29). In addition these studies had been carried out on patients who had received HSCT as treatment for malignancy. Both of these groups suggest that an early recovery of the TCR repertoire diversity is likely to be linked with lower risks of both GvHD and relapse, highlighting the clinical need to utilize this technology to monitor patients following treatment. As far as we are aware, this methodology has not yet been applied to pediatric CBT recipients, and therefore there is little knowledge on TCR clonotype composition and reconstitution dynamics in this cohort.

In this study, we found that TCR diversity in children who have received CBT is extremely heterogeneous both between and within individual patients, reflecting both the clinical background and the dynamics of the reconstitution process. Thirteen of the sixteen patients in this study are alive and well. Three patients died following disease relapse; patient A died 4 years post-CBT, patient C died 9 months post-CBT and patient J died 14 months post-CBT.

The repertoire typically tends to return to normality between 6 and 12 months after transplantation, although this trend needs to be confirmed in larger studies. We noticed one clear exception, patient O, who by month 12 had not yet reconstituted a diverse T cell receptor repertoire. This might be expected given that this patient relapsed with JMML within the first 3 months following CBT. They then received a subsequent CBT 6 months after their first transplant.

We observed considerable variance in diversity around month 1 post-CBT which is likely determined by a combination of three covariates: (1) the initial TCR repertoire in the patient, constituted by the cell pool the patient is left with following immune ablation, (2) the repertoire of the cord cell dose they are administered, and (3) the short-term reaction of this repertoire to the initial antigen landscape following transplantation. Interestingly, the TCR data demonstrated dramatic variation in the top 20 clonotypes expressed over time in patients post-CBT. These data contrast with the typically stable profile seen in healthy individuals, where comparatively little variation is present over time in the top clonotypes (31).

We show that the initial repertoire is unlikely to derive from recent thymic immigrants, since thymic output immediately following transplantation is low. As reconstitution continues, higher TREC levels correlate with an increased repertoire diversity, as captured by the low Gini coefficient scores, indicating thymic-dependent reconstitution of the naïve repertoire post-CBT (21). A plateau for thymic export of naïve T cells was observed between months 4 and 6, as reported previously (32).

CBT is increasingly used in preference to BMT, due in-part to the lower risk of acute and chronic GvHD. We observed that TCR diversity is inversely correlated to GvHD scores. Clinically, less diverse TCR profiles post-transplant may indicate the presence of large, dominant, donor-derived but host-directed clonotypes. There is significant interest in optimizing CD3+ cell dose in the cord blood unit for driving healthy reconstitution. We had hypothesized that CD3+ cell dose in the cord blood unit would impact on diversity following CBT, but found no clear relationship between these variables. Low patient numbers in combination with the inherent stochasticity in the colonizing process may be confounding any potential relationships. Studies with larger patient numbers across the range of CD3+ cell doses should be encouraged in order to investigate the impact of this variable further.

Several studies have investigated the impact on disease outcome between myeloablative (MAC) and reduced intensity (RIC) conditioning regimens ((33), the regimen's effect in transplantation-related morbidity, mortality, as well as tolerance in older patients (34)). However, no evaluation has ever been shown between conditioning and diversity in pediatric CBT recipients. In this study, no correlation between MAC/RIC and Gini coefficient was observed, indicating that the conditioning alone is not driving immune reconstitution following transplantation. The limitation of our patient group size emphasizes the need for larger cohorts so that the many confounders, together with conditioning, could be examined for their affect in disease outcome.

In addition to these clinical investigations, we investigated the clonal expansions seen in our CBT cohort by identifying antigen “specific” sequences within our samples, using VDJdb. The discovery of HIV-1 “specific” and clonally expanded sequences in clinically verified HIV seronegative samples supports the theory of high levels of TCR cross-reactivity across the repertoire. Given this finding, caution is advised over the use of the term “specific” in relationship to specific TCR sequences. An alternate and biologically appropriate method for communicating the reactivity of an individual's TCR profile would be to refer to a sequence or profile's “antigen binding potential.” Recent publications, such as Emerson et al. (35), have demonstrated that statistical signal is detectable in CDR3 sequences expressed in an individual's TCR repertoire, and using this have developed a classification framework for CMV diagnosis using immunosequencing of TCRβ molecules. Antigen specificity may therefore more usefully be considered a property of a repertoire, rather than of a set of specific sequences.

In conclusion, spectratyping remains the gold standard in clinical assessment of the TCR repertoire. We were able to verify the ability of NGS data to visualize clear immunophenotyping differences between clinically distinct, spectratyping-grouped patient samples. Furthermore, we were able to quantify this effect using diversity metrics. This is likely to be important in furthering our understanding of the complex confounding effects on immune recovery of conditioning regimen, source of hematological stem cells, cell doses, post-transplant prophyllaxis, GvHD, GvL, and underlying infection. We have now demonstrated a valuable new tool for quantitative assessment of T cell repertoire recovery that can utilized to improve our understanding of these effects. In summary our study provides strong rationale for further larger-scale prospective clinical trials, to establish the value of incorporating TCR repertoire sequencing as a routine prognostic or diagnostic test following pediatric hematological transplant.

## Author contributions

SA, ASG, BM, and RC conceived the study. SA, ASG, BM, JH, and TO designed the experiments. ASG, LM, and SA performed experiments and collected experimental data. MP, RC, and PV provided sample collection and collected clinical data. SA, ASG, BM, TA, JS, JB, NK, BC, and RC interpreted the data and wrote the manuscript. All authors approved the final version.

### Conflict of interest statement

The authors declare that the research was conducted in the absence of any commercial or financial relationships that could be construed as a potential conflict of interest.
