# Supplementary material for: Clinical T Cell Receptor Repertoire Deep Sequencing and Analysis: An Application to Monitor Immune Reconstitution Following Cord Blood Transplantation
Source: Front Immunol. 2018 Nov 5;9:2547. doi: 10.3389/fimmu.2018.02547 (PMC6231291; doi:10.3389/fimmu.2018.02547)
Supplement: Supplementary file 1 [file Data_Sheet_1.docx]

**Supplementary Material**

Due to the nature of the clinical samples collected for this project, extracted RNA quality and quantity often differed greatly, with varied input RNA concentrations potentially resulting in heterogenous sequencing depths between samples, prohibiting direct comparisons between samples. An initial analysis of the data (not presented here) suggested that arbitrary rarefication threshold selection led to misrepresentation of sample diversity and suboptimal sample inclusion rates. The heterogenous nature of the number of DCRs from each sample (range 353 – 189,048 sequences, median = 35,435) would mean that some would unavoidably need to be excluded from this analysis. The results from the algorithm developed to address this issue are presented below, where we were able to include 86 out of 146 samples at a subsampling depth of 10,232 sequences.

Supplementary figure 1 demonstrates the results from the subsampling algorithm, where the program can be seen to select points for rarefication at relatively low read depths, while maintaining separation between samples, as measured by diversity metrics. Lower subsampling depths than those selected exist past the visible asymptotes of many of the Gini rarefication curves, representing the points where the sample begins to loose structure and poorly represent its true diversity, potentially skewing the clinical interpretation of the result.

**Supplementary Figure 1. Results from the subsampling algorithm. (A) Plot of normalised Gini coefficient against percentage of subsampled read depth. The red cross on each line exhibits the minimum effective subsampling read depth for each sample, expressed as a percentage of the total sample read depth. (B) Raw Gini coefficient plotted against percentage of subsampled read depth as rarefication curves. Subsampling depths lower than those indicated on the plot will quickly tend to a Gini coefficient of 0 as the sample becomes even at an exponential rate relative to subsampling depth. (C) Representation of raw Gini coefficient plotted against raw subsampled read depth, showing the heterogeneity in number of DCRs extracted from the samples. (D) For comparison, normalised Shannon entropy versus percentage of subsampled read depth giving a similar result.**

| ID | Month | Chain | Num. Reads |
| --- | --- | --- | --- |
| A | 1 | a | 16848 |
| A | 2 | a | 74555 |
| A | 2 | b | 51850 |
| A | 3 | a | 31682 |
| A | 3 | b | 11997 |
| A | 6 | a | 63531 |
| A | 6 | b | 43617 |
| A | 12 | b | 55922 |
| B | 1 | a | 10992 |
| B | 1 | b | 15487 |
| B | 2 | a | 37021 |
| B | 2 | b | 34564 |
| C | 0 | b | 27223 |
| CBA | 1 | b | 17634 |
| CBB | 1 | a | 16088 |
| CBB | 1 | b | 29274 |
| CBC | 1 | b | 10712 |
| CBD | 1 | a | 10550 |
| CBD | 1 | b | 19874 |
| CBF | 1 | a | 11994 |
| CBF | 1 | b | 18451 |
| D | 1 | a | 14027 |
| D | 1 | b | 41393 |
| E | 1 | a | 37318 |
| E | 1 | b | 87469 |
| F | 1 | a | 42397 |
| F | 3 | a | 10350 |
| F | 3 | b | 29098 |
| F | 19 | b | 72924 |
| G | 1 | a | 42089 |
| G | 1 | b | 53241 |
| G | 6 | a | 47545 |
| G | 6 | b | 63185 |
| H | 2 | a | 44154 |
| H | 2 | b | 45423 |
| H | 6 | a | 79946 |
| H | 6 | b | 125292 |
| H | 12 | a | 71222 |
| H | 12 | b | 78722 |
| H | 18 | a | 33729 |
| H | 18 | b | 121199 |
| I | 2 | a | 41849 |
| I | 2 | b | 41673 |
| I | 4 | a | 50699 |
| I | 4 | b | 49493 |
| I | 6 | a | 30502 |
| I | 6 | b | 27165 |
| I | 12 | a | 102772 |
| I | 12 | b | 83062 |
| J | 1 | a | 17985 |
| J | 1 | b | 32366 |
| K | 1 | a | 48006 |
| K | 1 | b | 47919 |
| K | 2 | a | 61157 |
| K | 3 | a | 61106 |
| K | 3 | b | 66345 |
| L | 1 | a | 46865 |
| L | 1 | b | 57771 |
| L | 2 | a | 65341 |
| L | 2 | b | 10865 |
| L | 3 | a | 26672 |
| L | 3 | b | 60309 |
| L | 12 | a | 36307 |
| L | 12 | b | 70381 |
| L | 22 | a | 70549 |
| L | 22 | b | 62077 |
| M | 1 | a | 30070 |
| M | 1 | b | 60165 |
| M | 2 | b | 18227 |
| M | 6 | b | 21944 |
| M | 12 | a | 39366 |
| M | 22 | a | 15459 |
| M | 22 | b | 68477 |
| N | 1 | a | 49319 |
| N | 1 | b | 37955 |
| N | 3 | b | 57811 |
| N | 12 | a | 55738 |
| O | 2 | a | 11679 |
| O | 2 | b | 25874 |
| O | 12 | a | 46051 |
| O | 12 | b | 42877 |
| P | 1 | a | 22797 |
| P | 1 | b | 24512 |
| P | 2 | a | 11214 |
| P | 2 | b | 17859 |
| P | 17 | b | 15972 |
| P | 30 | a | 22351 |
| P | 30 | b | 39053 |

**Supplementary Table 1. Number of processed TCR sequences per sample prior to subsampling. Samples that were excluded by the subsampling process are not included in this table, or in any analyses. Raw fastq files are available from the Short Read Archive (**[**https://www.ncbi.nlm.nih.gov/sra**](https://www.ncbi.nlm.nih.gov/sra)**) under accession number SRP136075.**
